# Role of Inflammatory and Coagulation Biomarkers in Distinguishing Placenta Accreta from Placenta Previa and Associated Hemorrhage

**DOI:** 10.3390/jcm14113884

**Published:** 2025-05-31

**Authors:** Gülay Balkaş, Şevki Çelen

**Affiliations:** Department of Perinatology, University of Health Sciences, Etlik Zübeyde Women’ s Health Care Training and Research Hospital, 06010 Ankara, Turkey; sevkicelen@yahoo.com

**Keywords:** coagulation parameters, inflammatory parameters, intraoperative blood loss, placenta accreta spectrum, placenta previa

## Abstract

**Objectives:** This study aimed to differentiate patients with placenta accreta spectrum (PAS) from those with placenta previa (PP) and to assess the association between preoperative inflammatory and coagulation parameters and intraoperative blood loss. **Methods:** In this retrospective case-control study, 545 pregnant women were enrolled and divided into five groups: control (*n* = 251), PP (*n* = 246), PP with accreta (PPA, *n* = 18), PP with increta (PPI, *n* = 27), and PP with percreta (PPP, *n* = 33). Preoperative serum levels of neutrophil-to-lymphocyte ratio (NLR), platelet-to-lymphocyte ratio (PLR), systemic immune-inflammation index (SII), systemic inflammation response index (SIRI), delta neutrophil index (DNI), prothrombin time, fibrin degradation products (FDPs), D-dimer, and activated partial thromboplastin time (APTT) were analyzed. **Results:** The PPP group demonstrated significantly higher values of FDP, D-dimer, NLR, PLR, SII, SIRI, and DNI, and lower APTT values compared to the other groups (*p* < 0.001). For predicting PAS, SIRI and DNI showed the highest diagnostic performance, each achieving 100% sensitivity and specificity, with optimal cut-off values of 2.01 and 2.45, respectively. For predicting intraoperative blood loss ≥1000 mL, PLR and SIRI exhibited the highest diagnostic accuracy, with optimal cut-off values of 122.5 (sensitivity 76.6%; specificity 72.6%) and 2.25 (sensitivity 73.4%; specificity 74.1%), respectively. **Conclusions:** FDP, D-dimer, NLR, PLR, SII, SIRI, and DNI may serve as valuable biomarkers for differentiating PP from PAS, thereby enhancing preoperative risk assessment and guiding surgical planning to improve maternal outcomes. Additionally, PT, D-dimer, FDP, NLR, and DNI were identified as significant independent predictors of intraoperative blood loss.

## 1. Introduction

Placenta previa (PP) is a pregnancy complication defined by the partial or complete coverage of the endocervical os by the placenta [1]. It is associated with significant maternal and fetal-neonatal complications, many of which result directly from maternal hemorrhage [2]. Among these, placenta accreta spectrum (PAS) disorders—characterized by abnormal placental adhesion or invasion into the uterine wall—are particularly severe and complex [3,4]. As both PP and PAS are independently associated with increased morbidity and mortality, their coexistence amplifies maternal risks, exacerbating adverse maternal outcomes compared with PAS alone [5]. The recent global increase in cesarean section (C-section) rates has significantly changed the epidemiology of both conditions, with the incidence of PP now reaching 5.6 per 1000 pregnancies and PAS occurring in 24% of women after a first C-section and up to 67% after four or more [2].

PAS disorders are classified histologically based on the depth of villous invasion: placenta accreta, in which the villi adhere to the superficial myometrium; placenta increta, in which the villi invade the myometrium; and placenta percreta, characterized by villous invasion throughout the entire uterine wall, often extending into adjacent organs [4]. These histological subtypes often coexist in the same specimen, with involvement ranging from focal areas to extensive regions [6].

Failure to diagnose PAS prenatally is associated with severe maternal complications, primarily due to the extensive surgical interventions required to manage associated hemorrhage. These complications include cesarean hysterectomy, disseminated intravascular coagulation, massive blood transfusion, sepsis, and injury to adjacent organs, with maternal mortality rates reaching up to 7% in some regions [7]. Timely and accurate prenatal diagnosis enables critical preoperative planning, including the determination of optimal timing and setting for delivery, preparation of necessary blood products, and coordination of a multidisciplinary team experienced in the management of PAS [8]. In addition, precise assessment of PAS severity plays a crucial role in guiding clinical management, particularly in differentiating between abnormally adherent placenta and abnormally invasive placenta, as these require markedly different treatment approaches. Invasive cases typically necessitate a hysterectomy with the placenta left in situ, whereas conservative approaches—such as manual placental removal and uterine preservation—may be considered in carefully selected cases of adherent PAS [9].

While ultrasound remains the primary imaging modality for the diagnosis of PAS [10], magnetic resonance imaging (MRI) is used in selected cases [11]. However, the accuracy of these imaging modalities remains uncertain and is highly dependent on physician expertise and image quality. Given the significant morbidity and mortality associated with PAS, there is an urgent need for simple, cost-effective, and widely accessible diagnostic tools, particularly in low- and middle-income countries where healthcare resources are limited and access to advanced diagnostic centers is restricted.

The precise molecular mechanisms underlying invasive placentation remain poorly understood, with proposed hypotheses including the primary absence of the decidua or basal plate, abnormal maternal vascular remodeling, inflammation in cesarean scar tissue, and excessive extravillous trophoblastic invasion [12]. Normally, placental invasion is restricted to the inner third of the myometrium by tightly regulated spatial and temporal mechanisms. However, in PAS, the placenta exhibits invasive behavior, proliferating and invading local structures in a manner similar to malignant tumors [13].

Neutrophil-to-lymphocyte ratio (NLR), platelet-to-lymphocyte ratio (PLR), systemic immune-inflammation index (SII), systemic inflammatory response index (SIRI), and delta neutrophil index (DNI), which can be easily calculated from complete blood cell (CBC) parameters, are recognized as systemic inflammatory response markers. These indices are widely used to predict morbidity and mortality in inflammation-related disorders and certain cancers [14,15,16]. The similarity between trophoblast invasion patterns and cancer cell behavior has prompted research into the potential role of these biomarkers in PAS diagnosis [14,15,16].

Moreover, recent studies suggest that impaired coagulation and fibrinolytic disorders may be involved in the pathophysiology of PAS, suggesting their association with the severity of PAS [17,18]. However, the predictive value of coagulation parameters—including prothrombin time, activated partial thromboplastin time (APTT), D-dimer, and fibrin degradation products (FDPs)—remains understudied in PAS.

We sought to evaluate the significance of systemic inflammatory indices and coagulation parameters in predicting PAS among patients with PP and in determining the severity of PAS. Furthermore, this study aimed to assess the utility of these markers in relation to intraoperative blood loss, thereby addressing a critical knowledge gap in the existing literature and contributing to a more comprehensive understanding of PAS.

## 2. Materials and Methods

### 2.1. Study Design and Eligibility Criteria

This retrospective case-control study was conducted in the Department of Perinatology at a tertiary care center from January 2015 to November 2022. Participants aged 18–45 years with singleton pregnancies were enrolled. All participants were admitted either for third-trimester follow-up or due to vaginal bleeding, subsequently being diagnosed with PP or classified as controls without PP, and all underwent C-section. Exclusion criteria included fetal abnormalities, fetal demise, multiple gestation, intrauterine growth restriction, preterm premature rupture of membranes, history of smoking, acute or chronic inflammatory conditions, and pregestational diseases such as nephropathy, pregestational diabetes, thyroid dysfunction, or hypertension. Additionally, patients referred from other healthcare facilities after delivery were excluded.

### 2.2. Data Collection

Medical records were reviewed from the hospital’s obstetric and pathology databases. Gestational age was determined based on the last menstrual period and confirmed by ultrasound. Demographic and clinical data—including maternal age, gestational age at delivery, history of C-section, history of uterine curettage or surgery, need for hysterectomy, estimated intraoperative blood loss, transfusion of blood products, duration of surgery, and length of hospital stay—were recorded. Preoperative CBC and coagulation parameters were analyzed in a centralized laboratory at our tertiary care center. Intraoperative blood loss was estimated by summing the volume collected in the suction canister and the difference in weight between dry and blood-soaked gauze and surgical towels, assuming that 1 g equals 1 mL [19].

### 2.3. Calculation of Inflammatory Indices

Inflammatory markers were calculated using the following parameters: NLR: neutrophil count/lymphocyte count; PLR: platelet count/lymphocyte count; SII: neutrophil count × platelet count/lymphocyte count; SIRI: neutrophil count × monocyte count/lymphocyte count; DNI: the leukocyte subfraction assayed in the MPO channel by cytochemical reaction—the leukocyte subfraction counted in the nuclear lobularity channel by the reflected light beam [20]. At this institution, DNI is a routinely available parameter included in the CBC.

### 2.4. Diagnosis of Placenta Accreta and Placenta Previa

All patients underwent abdominal and transvaginal ultrasound with color Doppler imaging using a Voluson E6 device (GE Healthcare, Milwaukee, WI, USA). Examinations were conducted with a 3.5 MHz transabdominal transducer—performed with a partially filled bladder to enhance visualization of the uterine serosa and bladder wall—and a 7 MHz transvaginal transducer. These procedures were performed by a maternal-fetal medicine specialist under the supervision of a professor of maternal–fetal medicine with 25 years of experience.

The diagnosis of PP was established by transvaginal ultrasound at 32 weeks’ gestation, defined as complete coverage of the cervical os or a placental edge within 20 mm of the os. The diagnosis was confirmed by repeat ultrasound examination three weeks later [21].

The diagnosis of PAS was established based on the ultrasound criteria defined by the European Working Group on Abnormally Invasive Placenta [22]. Gray-scale and color Doppler criteria included myometrial thinning (<1 mm), loss of the normal hypoechoic retroplacental (clear) zone, abnormal and increased placental lacunae, bladder wall interruption, placental bulge and/or focal exophytic mass, uterovesical and subplacental hypervascularity, placental lacunar feeding vessels, and bridging vessels. The final diagnosis of PAS was based on clinical evidence of placental invasion observed at delivery and/or confirmed by histopathological examination of hysterectomy specimens, in accordance with the International Federation of Gynecology and Obstetrics guidelines [23]. FIGO grade 1 (placenta accreta) was diagnosed clinically when the placenta failed to detach despite the use of synthetic oxytocin and gentle controlled cord traction, necessitating mechanical or surgical intervention due to severe bleeding from the implantation site during manual removal attempts.

### 2.5. Statistical Analysis

Data analysis was performed using SPSS version 26.0 (SPSS Inc., Chicago, IL, USA). Continuous variables were presented as mean ± standard deviation (SD) or as median with interquartile range (IQR), while categorical variables were expressed as percentages. Kruskal-Wallis and Mann-Whitney U tests were used to analyze continuous independent variables, and the chi-squared test was used to analyze categorical variables, with Bonferroni correction applied for multiple comparisons. Receiver operating characteristic (ROC) curve analysis was performed to determine cut-off values, sensitivity, and specificity, and the area under the curve (AUC) was calculated to assess diagnostic performance. The Youden index (J = sensitivity + specificity − 1) was used to identify the optimal cut-off point by maximizing the overall diagnostic accuracy of the test. Multiple linear regression analysis was performed to identify parameters associated with blood loss ≥1000 mL. A *p*-value of less than 0.05 was considered statistically significant.

The study was conducted in accordance with the principles of the Declaration of Helsinki and received ethical approval from the ethics committee of the hospital (No: 21.04.2022-05/32). Patient consent was waived due to the retrospective design of the study.

## 3. Results

The maternal characteristics and perinatal outcomes of the study participants are shown in Table 1. A total of 575 patients were enrolled and divided into five groups: Group 1 (control, *n* = 251), Group 2 (PP, *n* = 246), Group 3 (PP with placenta accreta (PPA), *n* = 18), Group 4 (PP with placenta increta (PPI), *n* = 27), and Group 5 (PP with placenta percreta (PPP), *n* = 33). As shown in Table 1, the PPP group had significantly higher mean maternal age, body mass index, number of previous C-sections, hysterectomy rates, surgical duration, intraoperative blood loss, and duration of hospital stay compared with the other groups (*p* < 0.001).

Table 2 presents a comparison of inflammatory and coagulation parameters between the groups. The PPP group had significantly higher NLR, PLR, SII, SIRI, DNI, FDP, and D-dimer values and significantly lower APTT values compared to the other groups (*p* < 0.001).

**Figure 1:**
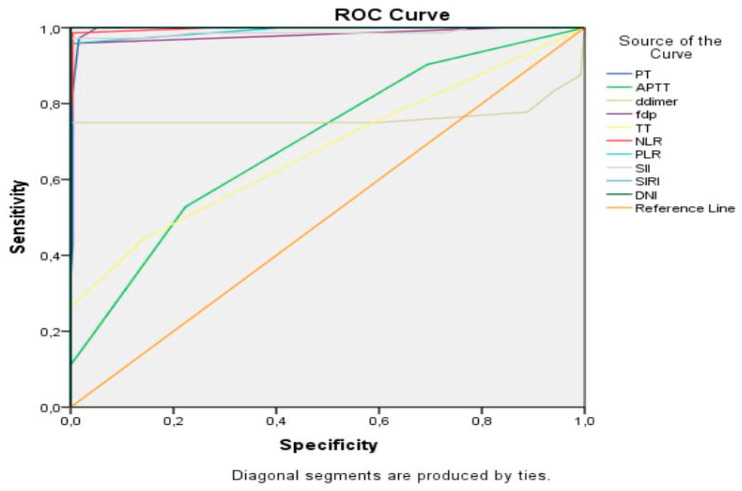
ROC curve of the inflammatory and coagulation parameters in predicting placenta accreta spectrum with placenta previa.

**Figure 2:**
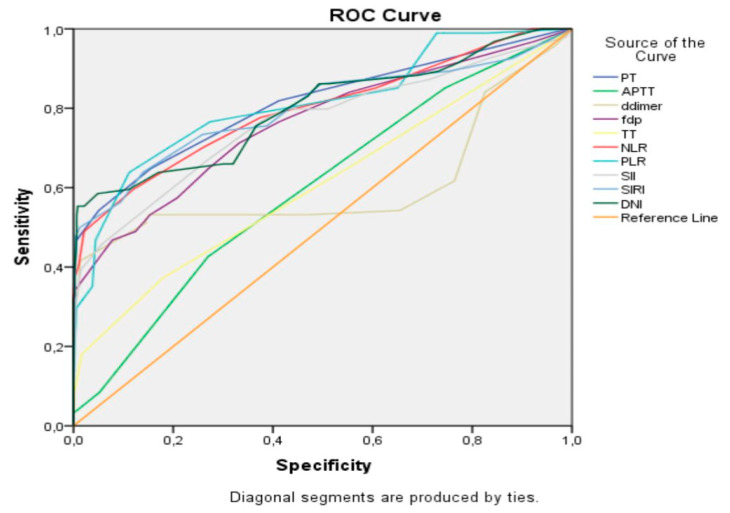
ROC curve of the inflammatory and coagulation parameters’ level in the estimate of ≥1000 mL intraoperative blood loss in the study population.

The predictive efficacy of the inflammatory and coagulation parameters for PAS and intraoperative blood loss ≥1000 mL was evaluated using ROC curve analysis (Figure 1 and Figure 2).

Among the markers evaluated, SIRI and DNI demonstrated the highest sensitivity and specificity for PAS (Table 3). The optimal cut-off value for SIRI, as determined by the Youden index, was 2.01, yielding 100% sensitivity and specificity (AUC: 1.00, 95% CI: 1.00–1.00, *p* < 0.001). Similarly, the optimal cut-off value for DNI was 2.45, also achieving 100% sensitivity and specificity (AUC: 1.00, 95% CI: 1.00–1.00, *p* < 0.001).

For predicting intraoperative blood loss ≥1000 mL, PLR and SIRI had the highest diagnostic accuracy (Table 4). The optimal cut-off value for PLR was 122.5, yielding a sensitivity of 76.6% and a specificity of 72.6% (AUC: 0.820, 95% CI: 0.739–0.855, *p* < 0.001). For SIRI, the optimal cut-off value was 2.25, with a sensitivity of 73.4% and a specificity of 74.1% (AUC: 0.798, 95% CI: 0.737–0.859, *p* < 0.001).

Multiple linear regression analysis was performed to evaluate the association between inflammatory and coagulation parameters, maternal characteristics, and intraoperative blood loss ≥1000 mL during C-section (Table 5). In Model A, PT, D-dimer, FDP, NLR, and DNI were significantly associated with increased blood loss. In Model B, after adjusting for established risk factors, PT, D-dimer, FDP, NLR, and DNI remained significant independent predictors of intraoperative hemorrhage.

## 4. Discussion

This study evaluated the predictive value of systemic inflammatory indices and coagulation parameters in patients with PP and PAS, as well as their association with increased intraoperative blood loss, yielding valuable findings for clinical practice. Maternal serum levels of NLR, PLR, SII, SIRI, DNI, D-dimer, FDP, and PT were significantly increased, whereas APTT levels were decreased in patients with PP and PAS compared to non-previa controls. Furthermore, after adjustment for established risk factors, PT, D-dimer, FDP, NLR, and DNI were identified as significant predictors of increased intraoperative blood loss. To our knowledge, this is the first study to investigate the predictive utility of inflammatory markers, particularly NLR and DNI, in relation to blood loss in cases of PP complicated by PAS. These findings highlight the potential role of these biomarkers in detecting PAS in PP cases, especially in resource-limited settings lacking advanced imaging or specialized expertise.

The increasing frequency of C-sections worldwide in recent decades has transformed PAS from a rare obstetric condition into a major public health challenge. PAS is one of the most life-threatening obstetric complications due to the severe maternal morbidity—including massive hemorrhage and peripartum hysterectomy—necessitated by surgical efforts to manage bleeding during delivery [6]. The risk of massive hemorrhage in PAS is closely related to the depth of placental invasion into the myometrium, the extent of abnormal placental adherence, and extrauterine extension into adjacent structures such as the bladder wall or the parametrium [24]. Consistent with these risks, our study demonstrated the highest intraoperative blood loss in the PPP group (2908 ± 672 mL), with significantly higher perioperative transfusion rates compared to other groups (*p* < 0.001). These findings are consistent with a retrospective study that found a mean blood loss of 3000 mL in 77 women with PAS who underwent peripartum hysterectomy [25].

A meta-analysis showed that 83.7% of patients with preoperative diagnosis of PAS required hysterectomy [26], whereas a study of 122 patients with PP without PAS reported a hysterectomy rate of only 0.8% [27]. Similarly, our study found a hysterectomy rate of 65.4% in the PAS group, significantly higher than the 4.0% observed in the PP-without-PAS group (*p* < 0.001). Therefore, preoperative differentiation between PAS and PP is crucial to optimize maternal outcomes, as delivery in specialized tertiary care centers with multidisciplinary teams significantly reduces maternal mortality from 7% in undiagnosed PAS cases to 0.05% [28]. Currently, ultrasound is the preferred modality for prenatal diagnosis and risk stratification of PAS due to its affordability, accessibility, and high diagnostic accuracy [10]. However, MRI is recognized as a complementary method, particularly in cases of posterior placenta, maternal obesity, or deep trophoblastic invasion [11]. Despite advances in prenatal diagnosis using ultrasound and MRI, limited access to tertiary care centers—particularly in low- and middle-income countries with limited healthcare resources—means that between half and two-thirds of PAS cases remain undiagnosed [29].

A combined approach integrating clinical assessment, established risk factors, imaging modalities, and emerging biomarkers may improve the accuracy of prenatal diagnosis of PAS. In recent years, maternal serum biomarkers such as pregnancy-associated plasma protein-A, alpha-fetoprotein, cell-free fetal DNA, and cell-free placental mRNA have been evaluated for their potential to improve diagnostic accuracy and to assess the severity of invasion in PAS cases [13]. However, not all biomarkers are routinely measurable in all clinical settings and there is a critical need for cost-effective, widely available serum-based tests to facilitate early identification of women at risk of PAS. NLR, PLR, SIRI, SII, and DNI—readily derived from CBC parameters—are widely used for the diagnosis, prognosis, and assessment of treatment response in both acute and chronic inflammatory conditions [15]. These biomarkers have also demonstrated prognostic value and sensitivity in several malignancies, including breast, ovarian, and colorectal cancer [30,31]. Their main advantage is cost-effectiveness, as they can be obtained through routine blood tests without the need for specialized equipment or advanced laboratory infrastructure.

Studies have identified distinct biological alterations in PAS placentas in which trophoblasts exhibit an abnormally invasive phenotype, possibly driven by a localized immunosuppressive microenvironment [32]. These changes are thought to be due to defective or absent decidua, often caused by previous uterine injury, which disrupts the normal regulatory mechanisms of trophoblast invasion [23]. Additionally, chronic basal inflammation is thought to contribute to both the progression and dysregulation of trophoblast invasion at the maternal–fetal interface [13]. In severe cases, the invasive placentation may extend into adjacent structures such as the urinary bladder or parametrium, exhibiting behavior reminiscent of malignant tumors [33]. Furthermore, studies have observed similarities between the pathophysiology of PAS and the tumor biology microenvironment, including disruption of local immune responses, evasion of growth regulatory pathways, and increased angiogenesis [34]. Given these similarities and the established prognostic value of CBC-derived parameters in various cancers, several studies have investigated the potential association between these parameters and placental invasion anomalies.

Yayla et al. investigated the predictive value of CBC parameters in placental invasion anomalies and grouped PP cases with and without invasion anomalies and reported that mean platelet volume (MPV), PLR, and NLR values were significantly increased, while red cell distribution width (RDW) was decreased in the invasion group [35]. Ersoy et al. compared PP cases with a control group and reported that, in the third trimester, total leukocyte count, neutrophil count, and NLR value were significantly higher, whereas MPV and large platelet cell ratio (P-LCR) were significantly lower in the PP group compared to controls [36]. However, patients who were diagnosed postnatally with PPP exhibited lower MPV and P-LCR values compared to other patients with PP [36]. Tasgoz et al. reported an increase in NLR and PLR in PP cases; however, they did not find significant differences in platelet distribution width (PDW) and RDW values [37]. A study by Karakoc et al. found no significant differences in NLR and PLR values between PP and PAS cases; however, DNI was significantly higher in patients with PAS [38]. In a case-control study, Keles et al. investigated the diagnostic utility of SII and other inflammatory markers in PAS, identifying significant differences in SII, PDW, MPV, NLR, and PLR between PP and PAS groups [39].

In this study, platelet and neutrophil counts were significantly elevated, while lymphocyte counts were reduced in patients with PAS, particularly in those with PPP. It is well established that platelets and neutrophils accumulate at sites of active inflammation, whereas lymphocytes serve as key regulators of the immune response. Neutrophils and platelets contribute to extracellular matrix remodeling by secreting proteases and cytokines, thereby facilitating angiogenesis and neovascularization [40,41]. Conversely, lymphocytes mediate cytotoxic cell death and suppress cellular proliferation and migration [42]. A reduction in lymphocyte count may disrupt immune regulation at the maternal–fetal interface, potentially contributing to abnormal placental invasion.

In the early phase of infection, excessive cytokine and chemokine production may interfere with neutrophil migration to the site of infection, resulting in a compensatory increase in neutrophil proliferation and the appearance of immature granulocytes in the peripheral blood. Nahm et al. introduced DNI, defined as the proportion of immature granulocytes among myeloperoxidase-positive cells [43]. A meta-analysis conducted by Park et al. assessed the diagnostic and prognostic utility of DNI, reporting a sensitivity of 67% and a specificity of 94% for detecting infections [20]. Consistent with these findings, elevated DNI values observed in our study corroborate the molecular mechanisms underlying acute and chronic inflammation implicated in the etiopathogenesis of PAS.

Our study demonstrated that alterations in the preoperative coagulation profile—specifically elevated PT, FDP, and D-dimer levels—were significantly associated with an increased risk of PPP. These findings suggest that excessive trophoblast invasion in PAS may lead to the depletion of coagulation factors and activation of fibrinolytic signaling pathways, resulting in hypocoagulability and hyperfibrinolysis. In support of this hypothesis, Jauniaux et al. identified fibrinolytic hyperactivity in the placental pathology of PAS, characterized by extensive fibrinoid deposition on the basal plate in regions of abnormally adherent and invasive villous tissue [18]. They proposed that these fibrinoid deposits result from abnormal maternal vascular remodeling in PAS, marked by dilatation of the radial and arcuate arteries. Such remodeling increases blood flow velocity and volume in the intervillous space, generating chronic shear forces that promote fibrin deposition, subsequent fibrin consumption, and dysregulated fibrinolytic activity [18].

Consistent with these findings, Guo et al. reported that elevated preoperative PT and FDP were significantly associated with an increased risk of FIGO grade 3 PAS and intraoperative blood loss volume [44]. Similarly, a study by Kong et al. showed that DNI could serve as a predictor of the need for massive transfusion after PPH [45]. In line with these findings, the present study identified PT, D-dimer, FDP, neutrophil-to-lymphocyte ratio (NLR), and DNI as significant independent predictors of increased intraoperative blood loss, even after adjustment for established risk factors.

Given the substantial maternal morbidity and mortality associated with PAS, there is a critical need for diagnostic strategies that are practical, resource-efficient, and broadly implementable—especially in low- and middle-income countries, where access to advanced imaging modalities and specialized care centers is often limited. The findings of this study contribute to addressing this gap by supporting the use of readily available clinical and laboratory parameters to aid in the early identification of high-risk patients. Such early detection may facilitate timely referral to tertiary care centers equipped with multidisciplinary teams experienced in PAS management, thereby improving maternal and neonatal outcomes.

The inclusion of novel inflammatory indices, the comprehensive assessment of numerous hematological parameters and a robust sample size are major strengths of this study. In addition, the management of all PAS cases by the same multidisciplinary team minimized potential bias related to operator expertise and surgical technique.

However, this study has several limitations. It was a retrospective study at a single center and blood samples were collected exclusively in the third trimester prior to surgery. Future prospective multicenter studies are warranted to validate these findings, evaluate the integration of inflammatory and coagulation biomarkers with imaging and clinical risk models for broader clinical application, and elucidate their temporal dynamics across the trimesters of pregnancy.

## 5. Conclusions

This study highlights the potential utility of inflammatory and coagulation biomarkers—specifically NLR, PLR, SII, SIRI, DNI, D-dimer, FDP, and PT—in differentiating PAS from PP. In addition, PT, D-dimer, FDP, NLR, and DNI were found to be significant independent predictors of intraoperative blood loss. These biomarkers may serve as valuable tools for early risk stratification, particularly in resource-limited settings with limited access to advanced imaging and specialized care.

## Figures and Tables

**Table 1:** Maternal characteristics and perinatal outcomes.

	Control (*n* = 251)	Placenta Previa (*n* = 246)	Previa and Accreta (*n* = 18)	Previa and Increta (*n* = 27)	Previa and Percrata (*n* = 33)	*p*
Maternal age (year) (mean ± SD)	25.5 ± 2.3	27.4 ± 1.7	29 ± 3.5	30.1 ± 4.4	34.5 ± 5.3	<0.001 *
BMI (kg/m^2^) (mean ± SD)	27.1 ± 0.8	27.3 ± 1.2	27.7 ± 1.3	28.2 ± 1.6	29.5 ± 1.8	<0.001 *
Gravidity ≥ 3 (*n*, %)	129 (51.4%) ^a^	137 (55.7%) ^a^	12 (66.6%) ^a^	27 (100%) ^b^	33 (100%) ^b^	<0.001
Parity ≥ 2 (*n*, %)	56 (22.3%) ^a^	77 (31.3%) ^a^	6 (33.3%) ^a^	27 (100%) ^b^	33 (100%) ^b^	<0.001
Number of C-sections (*n*, %)						<0.001
1	25 (10%) ^a^	128 (52%) ^b^	2 (11.1%) ^a^	0 (0%) ^a^	0 (0%) ^a^	
2	0 (0%) ^a^	79 (32.1%) ^b^	10 (55.5) ^b^	8 (29.6%) ^b^	0 (0%) ^a^	
3	0 (0%) ^a^	39 (15.9%) ^b^	5 (27.8%) ^b^	13 (48.2%) ^c^	22 (66.7%) ^c^	
4	0 (0%) ^a^	0 (0%) ^a^	1 (5.6%) ^a^	6 (22.2%) ^b^	11 (33.3%) ^b^	
History of abortion (*n*, %)	15 (6) ^a^	26 (10.6) ^a,b^	5 (27.7) ^b,c^	14 (51.8) ^c^	18 (54.5) ^c^	<0.001
Prior uterine curettage (*n*, %)	13 (5.2) ^a^	37 (15) ^b^	5 (27.7) ^b,c^	11 (40.7) ^c^	13 (39.4) ^c^	<0.001
Delivery week (mean ± SD)	39.2 ± 0.7	36.4 ± 0.9	37.1 ± 1.1	34.7 ± 0.6	34.6 ± 0.9	<0.001 *
Birth weight (g) (mean ± SD)	3184 ± 100	2795 ± 182	2759 ± 202	2419 ± 79	2382 ± 124	<0.001 *
Hysterectomy (*n*, %)	0 (0.0%) ^a^	1 (0.4%) ^a^	0 (0.0%) ^a^	18 (66.7%) ^b^	33 (100%) ^c^	<0.001
Operation time (min) (mean ± SD)	35.5 ± 5.7	52.3 ± 8.7	73.7 ± 8.0	99.2 ± 12.4	162.9 ± 27.5	<0.001 *
Blood loss (mL) (mean ± SD)	433.4 ± 176.1	851.6 ± 147.4	910 ± 135.4	1348 ± 193.3	2908.0 ± 672.0	<0.001 *
Blood transfusion (*n*, %)	3 (1.2%) ^a^	73 (29.7%) ^b^	17 (94.4%) ^c^	24 (88.9%) ^c^	33 (100%) ^c^	<0.001
Length of stay (day), (mean ± SD)	2.1 ± 0.3	2.3 ± 0.7	3.3 ± 0.6	4.4 ± 1.3	8.1 ± 1.0	<0.001 *

Abbreviations: BMI: body mass index; C-section: cesarean section; SD: standard deviation; * Kruskal-Wallis (Mann-Whitney U test) chi-squared test statistics; *p* < 0.05 compared with the control groups; ^a^ Kruskal-Wallis; ^b^ Mann-Whitney; ^c^ chi-squared.

**Table 2:** Analysis of preoperative inflammatory and coagulation parameters among the groups.

	Control ^1^	Previa ^2^	Previa and Accreta ^3^	Previa and Increta ^4^	Previa and Percrata ^5^	*p*	p *
PT (s) median (IQR)	10.1 (9.8–10.3)	10.2 (9.9–10.5)	10.3 (10.1–10.4)	10.5 (9.8–10.6)	11.1 (10.9–11.3)	<0.001	5 > 4 > 3 > 2 > 1
APTT (s) median (IQR)	26.6 (25.6–27.1)	26.5 (25.7–26.8)	26.4 (25.8–27.9)	26.3 (25.4–26.6)	25.9 (24.2–27.8)	0.024	1, 2, 3, 4 > 5
TT (s) median (IQR)	13.1 (12.5–14.1)	13.1 (12.8–13.7)	13.0 (12.6–13.8)	13.1 (12.4–14.1)	13.2 (12.4–14.8)	0.015	5 > 1, 2, 3, 4
Neutrophil (×10^9^/L) (mean ± SD)	8.3 ± 0.5	8.4 ± 0.7	8.5 ± 0.3	8.9 ± 0.2	9.2 ± 0.2	<0.001	5, 4 > 1, 2, 3
Lymphocyte (×10^9^/L) (mean ± SD)	1.9 ± 0.3	1.7 ± 0.8	1.6 ± 0.5	1.5 ± 0.6	1.5 ± 0.9	<0.001	1 > 2, 3 > 4, 5
Platelet (×10^9^/L) (mean ± SD)	207.5 ± 76.4	223.9 ± 62.8	216.9 ± 10.6	227.2 ± 10.9	282.3 ± 13.6	<0.001	5 > 4 > 2 > 3 > 1
D-dimer (mg/L) median (IQR)	0.44 (0.33–0.55)	0.42 (0.35–0.62)	0.46 (0.39–0.72)	0.65 (0.58–0.74)	1.05 (0.85–1.35)	<0.001	5 > 4 > 1, 2, 3
FDP (mg/L) median (IQR)	3.15 (2.15–4.52)	3.65 (2.84–4.85)	3.55 (2.90–5.15)	3.75 (2.64–4.94)	4.85 (3.55–7.85)	<0.001	5 > 2, 3, 4 > 1
NLR (mean ± SD)	3.68 ± 0.1	3.94 ± 0.1	4.07 ± 0.2	4.2 ± 0.1	4.9 ± 0.2	<0.001	5 > 3, 4 > 1, 2
PLR (mean ± SD)	116.6 ± 3.8	123.0 ± 7.1	128.4 ± 6.4	135.7± 6.1	141 ± 5.8	< 0.001	5 > 4 > 2, 3 > 1
SII (mean ± SD)	766.92 ± 33.73	869.11 ± 54.12	870.31 ± 52.61	970 ± 94.65	1122.58 ± 92.05	<0.001	5 > 4 > 2, 3 > 1
SIRI (mean ± SD)	1.62 ± 0.08	2.2 ± 0.21	2.41 ± 0.11	2.76 ± 0.23	3.29 ± 0.32	<0.001	5 > 4 > 3 > 2 > 1
DNI (mean ± SD)	1.8 ± 0.25	3.01 ± 0.25	3.34 ± 0.39	6.27 ± 0.56	8.74 ± 0.6	<0.001	5 > 4 > 2, 3 > 1

Abbreviations: PT: prothrombin time; APTT: activated partial thromboplastin time; TT: thrombin time; FDP: fibrin degradation product; NLR: neutrophil-to-lymphocyte ratio; PLR: platelet-to-lymphocyte ratio; SII: systemic immune-inflammation index; SIRI: systemic inflammation response index; DNI: delta neutrophil index; IQR: interquartile range; SD: standard deviation; Kruskal-Wallis * (Mann-Whitney U test) statistics; *p* < 0.05 compared with the control groups.

**Table 3:** Receiver operating characteristic (ROC) analysis of inflammatory and coagulation parameters for predicting placental invasion abnormalities with placenta previa.

	Cut-Off Value	Sensitivity	Specificity	AUC	95%CI	*p*
PT	10.25	0.972	0.984	0.996	0.991–1.00	<0.001
APTT	26.15	0.528	0.777	0.699	0.63–0.769	<0.001
D-dimer	0.445	0.750	0.669	0.764	0.669–0.859	<0.001
FDP	3.3	0.959	1.00	0.982	0.959–1.00	<0.001
TT	13.05	0.736	0.434	0.677	0.599–0.756	<0.001
NLR	3.85	0.986	0.996	0.997	0.992–1.00	<0.001
PLR	122.5	0.958	1.00	0.991	0.98–1.00	<0.001
SII	817.5	0.972	0.92	0.987	0.966–1.00	<0.001
SIRI	2.01	1.00	1.00	1.00	1.00–1.00	<0.001
DNI	2.45	1.00	1.00	1.00	1.00–1.00	<0.001

Abbreviations: PT: prothrombin time; APTT: activated partial thromboplastin time; TT: thrombin time; FDP: fibrin degradation product; NLR: neutrophil-to-lymphocyte ratio; PLR: platelet-to-lymphocyte ratio; SII: systemic immune-inflammation index; SIRI: systemic inflammation response index; DNI: delta neutrophil index.

**Table 4:** Receiver operating characteristic (ROC) analysis of inflammatory and coagulation parameters for predicting blood loss ≥ 1000 mL during surgery among pregnant women in the study.

	Cut-Off Value	Sensitivity	Specificity	AUC	95%CI	*p*
PT	10.25	0.649	0.844	0.811	0.754–0.868	<0.001
APTT	26.15	0.426	0.731	0.598	0.535–0.661	0.003
D-dimer	0.45	0.532	0.528	0.605	0.522–0.688	0.001
FDP	3.45	0.713	0.667	0.761	0.701–0.821	<0.001
TT	13.05	0.66	0.436	0.607	0.539–0.676	0.001
NLR	3.95	0.702	0.739	0.797	0.739–0.855	<0.001
PLR	122.5	0.766	0.726	0.820	0.739–0.855	<0.001
SII	875	0.67	0.731	0.763	0.739–0.855	<0.001
SIRI	2.25	0.734	0.741	0.798	0.737–0.859	<0.001
DNI	2.95	0.66	0.68	0.799	0.741–0.857	<0.001

Abbreviations: PT: prothrombin time; APTT: activated partial thromboplastin time; TT: thrombin time; FDP: fibrin degradation product; NLR: neutrophil-to-lymphocyte ratio; PLR: platelet-to-lymphocyte ratio; SII: systemic immune-inflammation index; SIRI: systemic inflammation response index; DNI: delta neutrophil index.

**Table 5:** Multiple linear regression analysis of parameters for blood loss ≥1000 mL.

	Model A		Model B	
	*β*	*p*	*β*	*p*
PT	0.142	0.001	0.153	<0.001
APTT	0.015	0.44		
D-dimer	0.067	0.026	0.066	0.027
FDP	0.229	<0.001	0.233	<0.001
TT	0.016	0.408		
NLR	0.141	0.001	0.148	0.001
PLR	0.022	0.433		
SII	0.043	0.221		
SIRI	−0.007	0.878		
DNI	0.292	<0.001	0.299	<0.001
Maternal age	0.03	0.227		
BMI	0,021	0.323		
Gravidity ≥ 3	−0.024	0.216		
Parity ≥ 2	−0.008	0.719		
Number of C-sections	0.086	0.012		
Abortus	−0.007	0.749		
D&C	−0.011	0.603		
Gestational age	0.055	0.092		
	F = 135.172		F = 240.442	
	R^2^ = 0.816		R^2^ = 0.812	

Abbreviations: PT: prothrombin time; APTT: activated partial thromboplastin time; TT: thrombin time; FDP: fibrin degradation product; NLR: neutrophil-to-lymphocyte ratio; PLR: platelet-to-lymphocyte ratio; SII: systemic immune-inflammation index; SIRI: systemic inflammation response index; DNI: delta neutrophil index; BMI: body mass index; D&C: dilatation and curettage; C-section: cesarean section.

## Data Availability

The data supporting the findings of this study are available from the corresponding author (G.B.) upon reasonable request.
